# Associations between Blood Pressure Indices and Brachial–ankle Pulse Wave Velocity in Treated Hypertensive Adults: results from the China Stroke Primary Prevention Trial (CSPPT)

**DOI:** 10.1038/s41598-019-44740-z

**Published:** 2019-06-03

**Authors:** Lihua Hu, Yuanyuan Zhang, Xiao Huang, Yun Song, Xianhui Qin, Binyan Wang, Yan Zhang, Genfu Tang, Jianping Li, Ping Li, Huihui Bao, Yong Huo, Xiaoshu Cheng

**Affiliations:** 1 Department of Cardiovascular Medicine, The Second Affiliated Hospital of Nanchang University, Nanchang of Jiangxi, China; 2 National Clinical Research Study Center for Kidney Disease, State Key Laboratory for Organ Failure Research, Renal Division, Nanfang Hospital, Southern Medical University, Guangzhou, China; 3 Institute of Biomedicine, Anhui Medical University, Hefei, China; 4 Department of Cardiology and Heart Center, Peking University First Hospital, Beijing, China; 5 School of Health Administration, Anhui University, Hefei, China

**Keywords:** Interventional cardiology, Hypertension

## Abstract

Brachial–ankle pulse wave velocity (baPWV), as a marker of arterial stiffness, has been demonstrated to be associated with blood pressure (BP) and onset of hypertension. However, little information is available on the associations between baPWV and BP indices [systolic BP (SBP), diastolic BP (DBP), pulse pressure (PP), mean arterial pressure (MAP)] in treated hypertensive patients. We aimed to assess the associations between BP indices and baPWV. In this cross-sectional study, 14,598 hypertensive patients from China Stroke Primary Prevention Trial (CSPPT) at the exit visit of the trial were analyzed. Elevated baPWV was defined as ≥18.3 m/s. Multivariate linear and logistic regression analyses were performed to evaluate the associations of BP indices with baPWV and elevated baPWV. Moreover, the smooth curve fitting (penalized spline method) was conducted. Multivariate linear regression analyses showed that continuous SBP, DBP, PP and MAP were independently and positively associated with baPWV (β = 0.081, 0.084, 0.078 and 0.115, respectively, all *P* < 0.001). Compared with controlled SBP group (<140 mm Hg), uncontrolled SBP (≥140 mm Hg) was significantly associated with higher baPWV [β = 2.234, 95% confidence interval (CI): 2.137–2.332]. Similarly, compared with controlled DBP group (<90 mm Hg), uncontrolled DBP (≥90 mm Hg) was significantly associated with higher baPWV (β = 1.466, 95%CI: 1.341–1.590). Multiple logistic analyses also showed that SBP, DBP, PP and MAP were significantly and positively associated with elevated baPWV (OR = 1.056, 1.049, 1.052, and 1.075, respectively, all *P* < 0.001). The fully-adjusted smooth curve fitting presented a linear association between BP indices with baPWV. In conclusion, among treated hypertensive patients, SBP, DBP, PP and MAP levels were independently and positively associated with baPWV and elevated baPWV, suggesting that baPWV might be a way to predict uncontrolled BP.

## Introduction

It is well-known that increased arterial stiffness is closely related to early vascular damage, cardiovascular disease (CVD) and mortality^1–3^. Brachial-ankle pulse wave velocity (baPWV) is available as a noninvasive measure of arterial stiffness^4,5^. The European Society of Hypertension (ESH) and of the European Society of Cardiology (ESC) recommended that baPWV should be a routine examination for hypertensive patients^6^. Hypertension is regarded as a public health challenge worldwide and is the leading modifiable risk factor for CVD^7,8^. Recent studies have showed that the control rate of hypertension is still low, although the awareness and treatment of hypertension have increased^9^. Poor blood pressure (BP) control further lead to target organ damage^10^. Therefore, it is important to identify the factors that influence individual response to anti-hypertensive treatment.

Indeed growing evidence has established the associations of baPWV with BP and onset of hypertension^11–16^. However, obvious conflicting results could be found among those reports. For instance, several epidemiological studies reported that BP indices was positively associated with baPWV^11–14^. In contrast, some studies showed that diastolic BP (DBP) was not associated with baPWV^15^. In addition, some studies indicated that DBP was negitvely associated with PWV^16^. These conflicting results might be attributed to the differences in cohort characteristics, sample size, and adjustment of confounders. Most of these previous studies have only discussed the association between BP and baPWV in general population. Uncertainty remains regarding the associations between BP indices [systolic BP (SBP), DBP, pulse pressure (PP), mean arterial pressure (MAP)] and baPWV because of limited data in treated hypertensive patients.

Therefore, our study aimed to assess the associations between BP indices and baPWV in a large sample of populations with treated hypertension.

## Methods

### Subject population and design

Our present study was a subset of the China Stroke Primary Prevention Trial (CSPPT)^17^. Briefly, the CSPPT is a large community-based, randomized, multicenter, double-blind, and actively controlled trial with a total of 20,702 hypertensive participants, conducted from May 2008 to August 2013 in Jiangsu and Anhui provinces of China. It was designed to confirm that enalapril maleate and folic acid tablets combined was more effective in preventing stroke among patients with hypertension when compared with enalapril maleate alone. Participants were scheduled for a visit (the first visit) after a 3-week run-in treatment period, and then were followed-up every 3 months until completion of the trial. Each visit involved recording BPs, heart rate, trial medication compliance, concomitant use of other medications, adverse events, and study outcome events. Details regarding inclusion/exclusion criteria, treatment assignment and outcome measures of the trial have been described in previous publications^17^ and shared on a related website (http://clinicaltrials.gov/ct2/show/NCT00794885). The study was approved by the ethics committee of the Nanfang Hospital, Guangzhou, China. The patients provided a written informed consent under the premises of the original CSPPT trial, including the possibility of post hoc analyses. This trial was registered with Clinicaltrials.gov (#NCT00794885). All methods were performed in accordance with the relevant guidelines and regulations.

At the exit visit of the trial, each participant was accpeted BaPWV measurement. In total, 20,702 eligible hypertensive participants were enrolled. Next, we selected participants according to the exclusion criteria listed below: missing baPWV values (n = 5504), missing BP values (n = 112), and ankle brachial index (ABI) < 0.90 (n = 488).

### Data collection

#### Brachial–ankle pulse wave velocity (baPWV)

BaPWV was automatically measured by PWV/ABI instruments (form PWV/ABI, BP-203RPE; Omron-Colin, Japan) as previously described by trained volunteers from medical colleges^18^. Briefly, occlusion and monitoring cuffs matched with oscillometric sensors were wrapped around subjects’ arms and the ankles, and pulse volume wave forms of the bilateral brachial and posterior tibial arteries were recorded simultaneously to determine the time interval between the initial increase in brachial and tibial waveforms (the transit time, Tba). The transmission distance from the brachium to ankle was calculated according to body height. The path length from the suprasternal notch to the brachium (Lb) was obtained using the following equation: Lb = 0.2159 × height of the patient (cm) − 2.0734. The path length from the suprasternal notch to the ankle (La) was obtained using the following equation: La = 0.8129 × height of the patient (cm) + 12.328. And the baPWV value was calculated as the ratio of transmission distance from the brachium to ankle divided by the transit time: baPWV = (La − Lb)/Tba. The maximum of the right and left-side baPWV values was used for analysis. The validation of this automatic device and its reproducibility have been previously published^2^.

#### BP and rest heart rate (RHR)

BP and RHR was measured at the last follow up visit with the subject in the sitting position after having rested for more than 5 minutes, and using an electronic sphygmomanometer (Omron; Dalian, China). Three consecutive measurements were obtained on the right arm, with 1-minute intervals between each. Then SBP, DBP, and RHR were calculated as the mean of three independent measures. MAP was calculated as [(2 × DBP) + SBP] / 3. PP was calculated as SBP – DBP. Uncontrolled SBP was defined as a SBP of ≥140 mm Hg and uncontrolled DBP was defined as a DBP of ≥90 mm Hg among treated hypertensive individuals.

#### Covariables

We selected these covariates on the basis of their associations with baPWV. Continuous variables included age (years), body mass index (BMI, kg/m^2^), serum homocysteine (hcy, μmol/L), fasting lipids [total cholesterol (TC, mmol/L), high-density lipoprotein-cholesterol (HDL-C, mmol/L), and triglycerides (TG, mmol/L)], fasting plasma glucose (FPG, mmol/L), creatinine (μmol/L), and uric acid (μmol/L). Categorical variables consisted of sex (male, female), Center (Lianyungang, Anqing), smoking (never smoking, former smoking or current smoking), alcohol consumption (never drinking, former drinking or current drinking), treatment group (enalapril, enalapril-folic acid), comorbidities (stroke, diabetes), medication use (combined with other antihypertensive drugs, lipid-lowering drugs and glucose-lowering drugs), methylenetetrahydrofolate reductase (*MTHFR)* C677T (rs1801133) polymorphism (CC, CT, TT). BMI was calculated as body weight in kilograms divided by the square of height in meters (kg/m^2^). Laboratory data were measured using automatic clinical analyzers (Beckman Coulter) at the core laboratory of the National Clinical Research Center for Kidney Disease, Nanfang Hospital, Guangzhou, China. *MTHFR* C677T polymorphisms were detected on an ABI Prism 7900HT sequence detection system (Life Technologies) using the TaqMan assay.

### Statistical analysis

Data are presented as mean ± SD for continuous variables and as frequency (%) for categorical variables. The population characteristics by baPWV quartiles were compared using ANOVA tests (continuous variables), or χ2 tests (categorical variables), accordingly. Taking BP indices (SBP, DBP, MAP, and PP) as independent variables and baPWV as an dependent variable, multivariate linear regression analyses were used to assess the β and 95% confidence interval (CI) of baPWV being associated with BP indices (SBP, DBP, MAP, and PP) adjustment for major covariables including age, sex, center, *MTHFR* C677T polymorphisms, treatment group, antihypertensive treatment, BMI, smoking status, alcohol consumption, RHR, TC, HDL-C, triglycerides, FPG, hcy, creatinine, uric acid. Meanwhile, Toshiaki Ohkuma *et al*.^19^ proposed that the optimal cutoff value of baPWV for cardiovascular disease in patients with hypertension was 18.3 m/s. Therefore, the elevated baPWV was defined as ≥18.3 m/s. We investigated the associations of BP indices with elevated baPWV using multivariate binary logistic regression analysis. BP indices were evaluated using models for both continuous and categorical variables according to clinical normal values and quartiles. The lowest level of each BP indice was considered as a reference group. To ensure the robustness of data analysis, we also did the sensitivity analyses. We performed tests for linear trend by entering the median value of each quartiles of BP indices as continuous variables in the models. Moreover, to further characterize the shape of the associations between BP indices and baPWV, we used the smooth curve fitting (penalized spline method). Interaction and stratified analyses were performed to evaluate whether covariates influenced the associations between BP indices and baPWV.

All the analyses were performed using the statistical package R (http://www.R-project.org, The R Foundation) and Empower (R) (www.empowerstats.com; X&Y Solutions, Inc., Boston, MA). A 2-tailed *P* < 0.05 was considered to be statistically significant.

## Results

### Characteristics of the subjects

**Figure 1:**
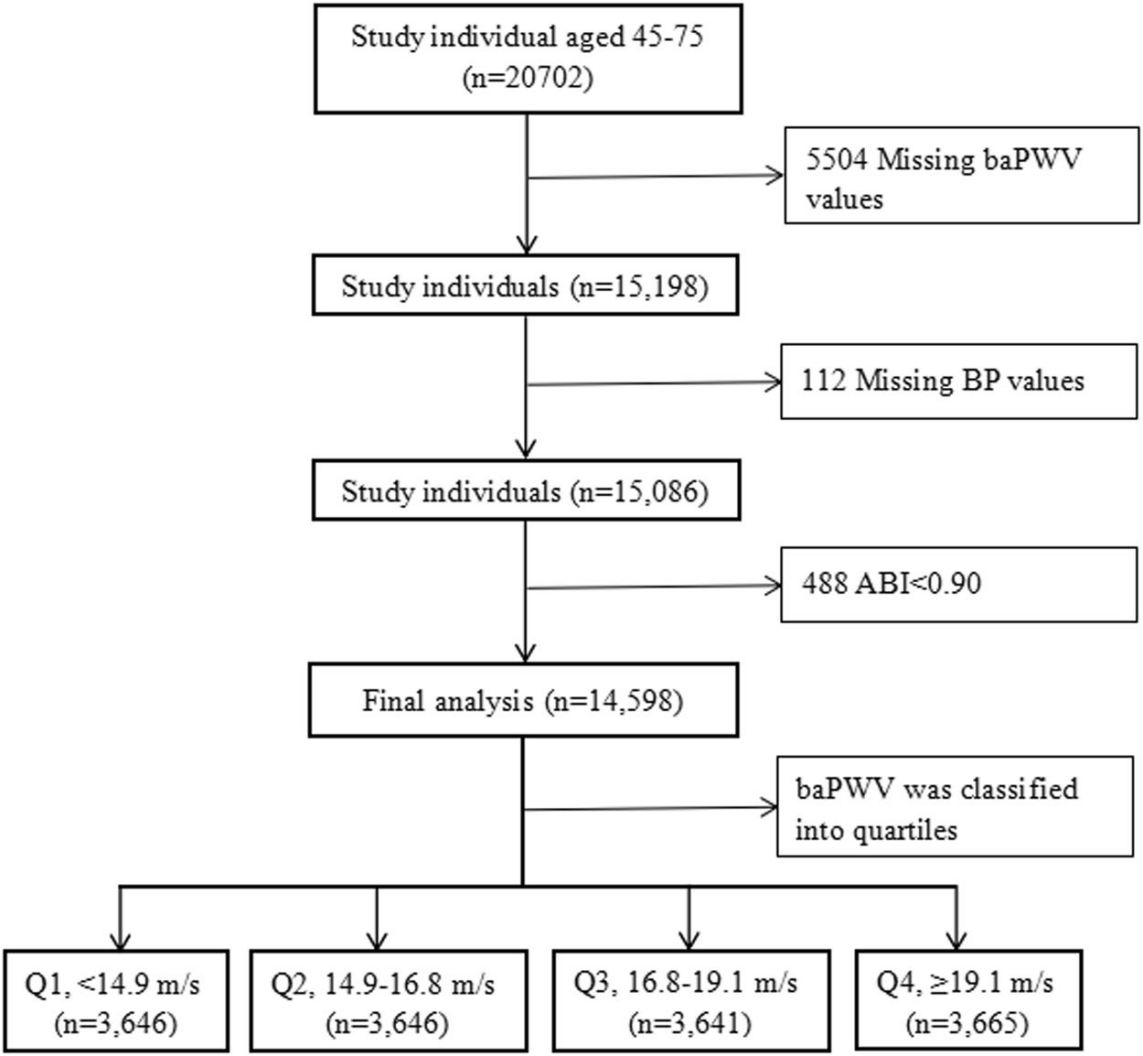
Flow chart of study participants. Abbreviations: baPWV, brachial–ankle pulse wave velocity; BP, blood pressure; ABI, ankle brachial index.

Based on the inclusion and exclusion criteria, a total of 14,598 participants (mean age: 64.4 ± 7.4 years; 40.2% males) at the exit visit were selected for final data analysis (Fig. 1). Of the participants, 5.1% had experienced a stroke and 2.3% had diabetes. The mean (SD) values for SBP, DBP, PP, and MAP were 135.4 (17.4) mmHg, 81.9 (10.9) mmHg, 53.5 (15.3) mmHg, and 99.7 (11.3) mmHg, respectively. The mean baPWV was 17.3 (3.4) m/s. The distributions of selective participants sociodemographic characteristics and other covariates according to baPWV quartiles (<14.9, 14.9–16.8, 16.8–19.1 and ≥19.1 m/s) are presented in Table 1. Compared with participants with baPWV in the lower quartiles (Q1–Q3), participants with baPWV in the highest quartile (Q4) had higher values in age, RHR, SBP, DBP, MAP, PP, FPG, TC, TG, hcy, uric acid, creatinine and higher rates of stroke, diabetes, antihypertensive and glucose-lowering drugs (all *P* < 0.01). In contrast, populations in the highest baPWV group had significantly low BMI value. No significant differences were found between the 4 groups in terms of sex, *MTHFR* genotype distribution, alcohol consumption, HDL-cholesterol, lipid-lowering drugs or treatment group (all *P* > 0.05).

**Table 1:** Baseline characteristics of study participants.

Characteristics^*^	Total	BaPWV quartiles, m/s				
		Q1(<14.9)	Q2(14.9–16.8)	Q3(16.8–19.1)	Q4(≥19.1)	*P* value
N (%)	14598	3646	3646	3641	3665	
Male, N (%)	5864 (40.2)	1494 (41.0)	1486 (40.8)	1459 (40.1)	1425 (38.9)	0.255
Age, years	64.4 ± 7.4	60.4 ± 6.7	63.1 ± 6.9	65.4 ± 6.8	68.6 ± 6.4	<0.001
BMI, kg/m^2^	24.9 ± 3.8	25.5 ± 3.9	25.1 ± 3.8	24.7 ± 3.8	24.4 ± 3.7	<0.001
RHR, bpm	76.8 ± 11.3	73.8 ± 10.1	75.5 ± 10.5	77.2 ± 11.1	80.8 ± 12.4	<0.001
baPWV, m/s	17.3 ± 3.4	13.5 ± 1.0	15.8 ± 0.6	17.8 ± 0.7	21.9 ± 2.6	<0.001
**Center, N (%)**						<0.001
Lianyungang	3497 (24.0)	689 (18.9)	869 (23.8)	927 (25.5)	1012 (27.6)	
Anqing	11101 (76.0)	2957 (81.1)	2777 (76.2)	2714 (74.5)	2653 (72.4)	
***MTHFR*** **C677T polymorphisms, N (%)**					0.182	
CC	3963 (27.1)	933 (25.6)	984 (27.0)	1028 (28.2)	1018 (27.8)	
CT	7144 (48.9)	1817 (49.8)	1781 (48.8)	1780 (48.9)	1766 (48.2)	
TT	3491 (23.9)	896 (24.6)	881 (24.2)	833 (22.9)	881 (24.0)	
**Smoking status, N (%)**						0.002
Never	9844 (67.7)	2483 (68.3)	2444 (67.2)	2462 (67.9)	2455 (67.3)	
Former	1641 (11.3)	371 (10.2)	375 (10.3)	447 (12.3)	448 (12.3)	
Current	3065 (21.1)	783 (21.5)	816 (22.4)	719 (19.8)	747 (20.5)	
**Alcohol consumption, N (%)**					0.201	
Never	9878 (70.2)	2454 (69.8)	2437 (69.5)	2498 (71.1)	2489 (70.5)	
Former	972 (6.9)	234 (6.7)	228 (6.5)	259 (7.4)	251 (7.1)	
Current	3219 (22.9)	829 (23.6)	839 (23.9)	758 (21.6)	793 (22.4)	
**BP indices, mm Hg**						
SBP	135.4 ± 17.4	126.2 ± 14.3	132.9 ± 14.8	137.2 ± 15.9	145.1 ± 18.5	<0.001
DBP	81.9 ± 10.9	81.2 ± 10.3	81.9 ± 10.6	81.7 ± 10.9	82.8 ± 11.6	<0.001
PP	53.5 ± 15.3	45.0 ± 12.3	51.1 ± 13.1	55.5 ± 14.0	62.2 ± 16.0	<0.001
MAP	99.7 ± 11.3	96.2 ± 10.2	98.9 ± 10.5	100.2 ± 11.0	103.6 ± 12.1	<0.001
**Laboratory results**						
FPG, mmol/L	6.2 ± 1.9	6.0 ± 1.5	6.1 ± 1.8	6.3 ± 2.0	6.5 ± 2.4	<0.001
TC, mmol/L	5.3 ± 1.1	5.2 ± 1.0	5.3 ± 1.1	5.3 ± 1.1	5.4 ± 1.1	<0.001
HDL-C, mmol/L	1.3 ± 0.3	1.3 ± 0.3	1.3 ± 0.3	1.3 ± 0.3	1.3 ± 0.3	0.477
TG, mmol/L	1.8 ± 1.5	1.7 ± 1.3	1.7 ± 1.3	1.8 ± 1.6	1.9 ± 1.5	<0.001
Hcy, μmol/L	13.5 ± 7.1	12.9 ± 7.9	13.2 ± 5.7	13.5 ± 6.0	14.4 ± 8.4	<0.001
Uric acid, μmol/L	329.3 ± 89.7	323.5 ± 85.8	328.3 ± 88.7	329.8 ± 90.5	335.4 ± 93.2	<0.001
Creatinine, μmol/L	68.7 ± 26.8	67.9 ± 24.6	68.2 ± 21.8	69.3 ± 34.8	69.5 ± 24.3	0.032
**Comorbidities**, **N (%)**						
Stroke	739 (5.1)	141 (3.9)	172 (4.7)	203 (5.6)	223 (6.1)	<0.001
Diabetes	333 (2.3)	44 (1.2)	83 (2.3)	83 (2.3)	123 (3.4)	<0.001
**Medications use, N (%)**						
Antihypertensive drugs	14137 (97.2)	3505 (96.3)	3542 (97.4)	3526 (97.2)	3564 (97.7)	0.007
Lipid-lowering drugs	212 (1.5)	58 (1.6)	62 (1.7)	45 (1.2)	47 (1.3)	0.343
Glucose-lowering drugs	830 (5.8)	133 (3.8)	189 (5.3)	239 (6.8)	269 (7.5)	<0.001
**Treatment group, N (%)**						0.258
Enalapril	7322 (50.2)	1791 (49.1)	1870 (51.3)	1808 (49.7)	1853 (50.6)	
Enalapril-folic acid	7276 (49.8)	1855 (50.9)	1776 (48.7)	1833 (50.3)	1812 (49.4)	

^*^Data are presented as number (%) or mean ± standard deviation.

Abbreviations: BMI = Body mass index; RHR = resting heart rate; BaPWV = Brachial-ankle pulse wave velocity; *MTHFR* = methylenetetrahydrofolate reductase; BP = blood pressure; SBP = systolic blood pressure; DBP = diastolic blood pressure; MAP = mean arterial pressure; PP = pulse pressure; FPG = fasting plasma glucose; TC = Total cholesterol; HDL-C = high density lipoprotein cholesterol; TG = triglycerides; Hcy = homocysteine.

### Association between BP indices and baPWV

**Figure 2:**
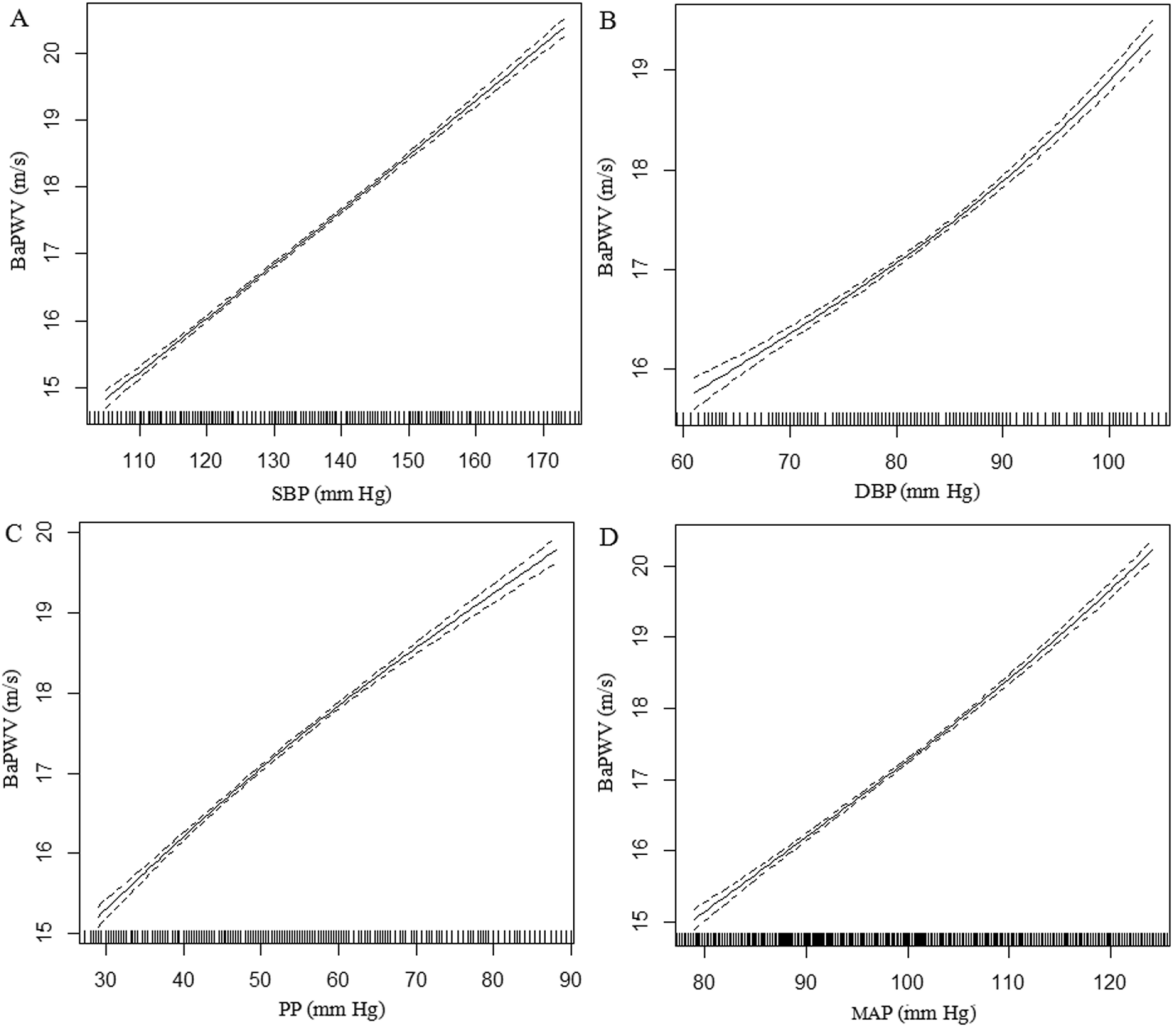
Association between BP indices and baPWV. (**A**) SBP and baPWV; (**B**) DBP and baPWV; (**C**) PP and baPWV; (**D**) MAP and baPWV. The smooth curve fitting presented linear associations between all BP indices and baPWV among participants with treated hypertension. The solid line and dashed line represent the estimated values and their corresponding 95% confidence intervals (CI). Abbreviations: baPWV, brachial–ankle pulse wave velocity; BP, blood pressure; SBP, systolic blood pressure; DBP, diastolic blood pressure; PP, pulse pressure; MAP, mean arterial pressure. *Adjustment factors included age, sex, center, MTHFR C677T polymorphisms, treatment group, antihypertensive treatment, BMI, smoking status, and alcohol consumption, RHR, TC, HDL-cholesterol, TG, FPG, creatinine, homocysteine, uric acid*.

The associations of baPWV with BP indices assessed by multivariate linear regression analyses are listed in Table 2. In the crude model, continuous SBP, DBP, PP and MAP were significantly and positively associated with baPWV (β = 0.081, 0.024, 0.093 and 0.079, respectively, all *P* < 0.001). In addition, after adjustment for all confounding factors including age, sex, center, *MTHFR* C677T polymorphisms, treatment group, antihypertensive treatment, BMI, smoking status, alcohol consumption, RHR, TC, HDL-C, TG, FPG, creatinine, hcy, and uric acid, all BP indices were independently and positively associated with baPWV (β = 0.081, 0.084, 0.078 and 0.115, respectively, all *P* < 0.001). BP indices were also evaluated for categorical variables according to clinical normal values and quartiles. Compared with controlled SBP group (<140 mm Hg), uncontrolled SBP (≥140 mm Hg) was significantly associated with higher baPWV (β = 2.234, 95%CI: 2.137–2.332, *P* < 0.001). Similarly, compared with controlled DBP group (<90 mm Hg), uncontrolled DBP (≥90 mm Hg) was significantly associated with higher baPWV (β = 1.466, 95%CI: 1.341–1.590, *P* < 0.001). Patients with higher PP levels (≥40 mmHg) had higher baPWV (β = 1.888, 95%CI: 1.754–2.022, *P* < 0.001). Compared with normal MAP levels (70–105 mmHg), high MAP levels (≥105 mmHg) were positively associated with baPWV (β = 2.080, 95%CI: 1.973–2.187, *P* < 0.001), while low MAP levels (<70 mmHg) were negatively associated with baPWV (β = −3.224, 95%CI: −4.235–2.212, *P* < 0.001). When BP indices were classified into quartiles, we observed a significant and progressive increase in baPWV with BP quartiles (all *P* for trend < 0.001), suggesting a dose-dependent increase in baPWV with BP indices. Further analyses using smooth curve fitting (penalized spline method) confirmed that the associations of BP indices with baPWV were linear (Fig. 2).

**Table 2:** Associations of Brachial-ankle pulse wave velocity with BP indices.

Variables	N	Mean ± SD	Crude model		Adjusted model^*^	
			β (95%CI)	*P* value	β (95%CI)	*P* value
**SBP, mmHg**						
Continuous	14598	17.3 ± 3.4	0.081 (0.078, 0.084)	<0.001	0.081 (0.079, 0.084)	<0.001
**Categories**						
<140	9091	16.4 ± 3.0	Reference (0)		Reference (0)	
≥140	5507	18.8 ± 3.5	2.367 (2.260, 2.474)	<0.001	2.234 (2.137, 2.332)	<0.001
**Quartiles**						
Q1 (<123.3)	3647	15.7 ± 2.8	Reference (0)		Reference (0)	
Q2 (123.3–134.0)	3533	16.6 ± 2.9	0.976 (0.831, 1.122)	<0.001	1.080 (0.950, 1.210)	<0.001
Q3 (134.0–145.3)	3664	17.6 ± 3.1	1.931 (1.787, 2.075)	<0.001	1.951 (1.822, 2.081)	<0.001
Q4 (≥145.3)	3754	19.2 ± 3.6	3.500 (3.357, 3.644)	<0.001	3.447 (3.317, 3.577)	<0.001
*P* for trend			<0.001		<0.001	
**DBP, mmHg**						
Continuous	14598	17.3 ± 3.4	0.024 (0.019, 0.029)	<0.001	0.084 (0.079, 0.089)	<0.001
**Categories**						
<90	11300	17.2 ± 3.3	Reference (0)		Reference (0)	
≥90	3298	17.7 ± 3.6	0.499 (0.367, 0.631)	<0.001	1.466 (1.341, 1.590)	<0.001
**Quartiles**						
Q1 (<74.7)	3483	17.1 ± 3.2	Reference (0)		Reference (0)	
Q2 (74.7–81.3)	3628	17.2 ± 3.3	0.055 (−0.103, 0.213)	0.495	0.650 (0.509, 0.791)	<0.001
Q3 (81.3–89.3)	3835	17.2 ± 3.4	0.109 (−0.047, 0.265)	0.171	1.138 (0.995, 1.282)	<0.001
Q4 (≥89.3)	3652	17.6 ± 3.6	0.513 (0.355, 0.670)	<0.001	2.139 (1.986, 2.293)	<0.001
*P* for trend			<0.001		<0.001	
**PP, mmHg**						
Continuous	14598	17.3 ± 3.4	0.093 (0.090, 0.096)	<0.001	0.078 (0.074, 0.081)	<0.001
**Categories**						
<40	2529	15.3 ± 2.6	Reference (0)		Reference (0)	
≥40	12069	17.7 ± 3.4	2.419 (2.278, 2.559)	<0.001	1.888 (1.754, 2.022)	<0.001
**Quartiles**						
Q1 (<42.7)	3640	15.5 ± 2.7	Reference (0)		Reference (0)	
Q2 (42.7–52.0)	3605	16.7 ± 3.1	1.223 (1.079, 1.367)	<0.001	1.013 (0.878, 1.148)	<0.001
Q3 (52.0–62.0)	3583	17.7 ± 3.2	2.229 (2.085, 2.373)	<0.001	1.826 (1.689, 1.964)	<0.001
Q4 (≥62.0)	3770	19.1 ± 3.5	3.628 (3.486, 3.771)	<0.001	2.922 (2.781, 3.063)	<0.001
*P* for trend			<0.001		<0.001	
**MAP, mmHg**						
Continuous	14598	17.3 ± 3.4	0.079 (0.074, 0.084)	<0.001	0.115 (0.110, 0.119)	<0.001
**Categories**						
<70	35	14.9 ± 2.5	−1.925 (−3.029,−0.820)	<0.001	−3.224 (−4.235, −2.212)	<0.001
70–105	10209	16.8 ± 3.2	Reference (0)		Reference (0)	
≥105	4354	18.3 ± 3.7	1.521 (1.403, 1.639)	<0.001	2.080 (1.973, 2.187)	<0.001
**Quartiles**						
Q1 (<92.2)	3624	16.4 ± 3.0	Reference (0)		Reference (0)	
Q2 (92.2–99.1)	3640	16.8 ± 3.2	0.474 (0.322, 0.626)	<0.001	0.918 (0.785, 1.051)	<0.001
Q3 (99.1–106.7)	3681	17.4 ± 3.3	1.076 (0.924, 1.227)	<0.001	1.712 (1.578, 1.847)	<0.001
Q4 (≥106.7)	3653	18.5 ± 3.7	2.127 (1.975, 2.279)	<0.001	3.101 (2.963, 3.240)	<0.001
*P* for trend			<0.001		<0.001	

Abbreviations: CI = confidence interval; BP = blood pressure; SBP = systolic blood pressure; DBP = diastolic blood pressure; MAP = mean arterial pressure; PP = pulse pressure.

^*^Adjusted for age, sex, center, MTHFR C677T polymorphisms, treatment group, antihypertensive treatment, BMI, smoking status, and alcohol consumption, RHR, TC, HDL-C, TG, FPG, creatinine, hcy, uric acid.

Table 3 shows the associations between BP indices and elevated baPWV. In adjusted model, SBP, DBP, PP and MAP were independently and positively associated with elevated baPWV in treated hypertensive patients [odds ratio (OR) = 1.056, 1.049, 1.052, and 1.075, respectively, all *P* < 0.001]. Compared with SBP control group, uncontrolled SBP was significantly associated with increased prevalence of elevated baPWV (OR = 4.514, 95%CI: 3.808–4.531, *P* < 0.001). Similarly, uncontrolled DBP was associated with increased prevalence of elevated baPWV (OR = 2.261, 95%CI: 2.038–2.508, *P* < 0.001) compared with DBP control group. Also, *P* for trend for all BP indices in the all models was significant, indicating that BP indices were positively associated with elevated baPWV.

**Table 3:** Associations between BP indices and elevated brachial-ankle pulse wave velocity

Variables	Events (%)	Crude model		Adjusted model^*^	
		OR (95%CI)	*P* value	OR (95%CI)	*P* value
**SBP, mmHg**					
Continuous	4739 (32.5)	1.045 (1.043, 1.048)	<0.001	1.056 (1.053, 1.059)	<0.001
**Categories**					
<140	2021 (22.2)	Reference (1)		Reference (1)	
≥140	2718 (49.4)	3.409 (3.171, 3.665)	<0.001	4.154 (3.808, 4.531)	<0.001
**Quartiles**					
Q1 (<123.3)	570 (15.6)	Reference (1)		Reference (1)	
Q2 (123.3–134.0)	860 (24.3)	1.737 (1.544, 1.954)	<0.001	2.111 (1.844, 2.417)	<0.001
Q3 (134.0–145.3)	1295 (35.3)	2.951 (2.638, 3.301)	<0.001	3.824 (3.354, 4.361)	<0.001
Q4 (≥145.3)	2014 (53.7)	6.248 (5.597, 6.975)	<0.001	9.364 (8.197, 10.698)	<0.001
*P* for trend		<0.001		<0.001	
**DBP, mmHg**					
Continuous	4739 (32.5)	1.008 (1.005, 1.011)	<0.001	1.049 (1.044, 1.053)	<0.001
**Categories**					
<90	3586 (31.7)	Reference (1)		Reference (1)	
≥90	1153 (35.0)	1.156 (1.065, 1.255)	<0.001	2.261 (2.038, 2.508)	<0.001
**Quartiles**					
Q1 (<74.7)	1109 (31.8)	Reference (1)		Reference (1)	
Q2 (74.7–81.3)	1144 (31.5)	0.986 (0.892, 1.089)	0.780	1.382 (1.229, 1.553)	<0.001
Q3 (81.3–89.3)	1226 (32.0)	1.006 (0.912, 1.110)	0.906	1.887 (1.675, 2.127)	<0.001
Q4 (≥89.3)	1260 (34.5)	1.128 (1.022, 1.245)	0.017	3.201 (2.811, 3.646)	<0.001
*P* for trend		0.016		<0.001	
**PP, mmHg**					
Continuous	4739 (32.5)	1.054 (1.052, 1.057)	<0.001	1.052 (1.048, 1.055)	<0.001
**Categories**					
<40	285 (11.3)	Reference (1)		Reference (1)	
≥40	4454 (36.9)	4.605 (4.049, 5.238)	<0.001	4.148 (3.584, 4.800)	<0.001
**Quartiles**					
Q1 (<42.7)	470 (12.9)	Reference (1)		Reference (1)	
Q2 (42.7–52.0)	900 (25.0)	2.244 (1.985, 2.537)	<0.001	2.140 (1.865, 2.457)	<0.001
Q3 (52.0–62.0)	1317 (36.8)	3.920 (3.483, 4.412)	<0.001	3.629 (3.173, 4.150)	<0.001
Q4 (≥62.0)	2052 (54.4)	8.056 (7.172, 9.048)	<0.001	6.953 (6.075, 7.957)	<0.001
*P* for trend		<0.001		<0.001	
**MAP, mmHg**					
Continuous	4739 (32.5)	1.038 (1.035, 1.042)	<0.001	1.075 (1.070, 1.080)	<0.001
**Categories**					
<70	4 (11.4)	0.333 (0.117, 0.943)	0.038	0.152 (0.050, 0.457)	<0.001
70–105	2854 (28.0)	Reference (1)		Reference (1)	
≥105	1881 (43.2)	1.960 (1.820, 2.111)	<0.001	3.407 (3.101, 3.743)	<0.001
**Quartiles**					
Q1 (<92.2)	826 (22.8)	Reference (1)		Reference (1)	
Q2 (92.2–99.1)	1032 (28.4)	1.340 (1.206, 1.490)	<0.001	1.885 (1.664, 2.134)	<0.001
Q3 (99.1–106.7)	1238 (33.6)	1.717 (1.548, 1.904)	<0.001	2.980 (2.630, 3.376)	<0.001
Q4 (≥106.7)	1643 (45.0)	2.769 (2.502, 3.064)	<0.001	7.013 (6.149, 7.998)	<0.001
*P* for trend		<0.001		<0.001	

Abbreviations: OR = odds ratio; CI = confidence interval; BP = blood pressure; SBP = systolic blood pressure; DBP = diastolic blood pressure; MAP = mean arterial pressure; PP = pulse pressure.

^*^Adjusted for age, sex, center, MTHFR C677T polymorphisms, treatment group, antihypertensive treatment, BMI, smoking status, and alcohol consumption, RHR, TC, HDL-C, TG, FPG, creatinine, hcy, uric acid.

### Subgroup analysis

**Figure 3:**
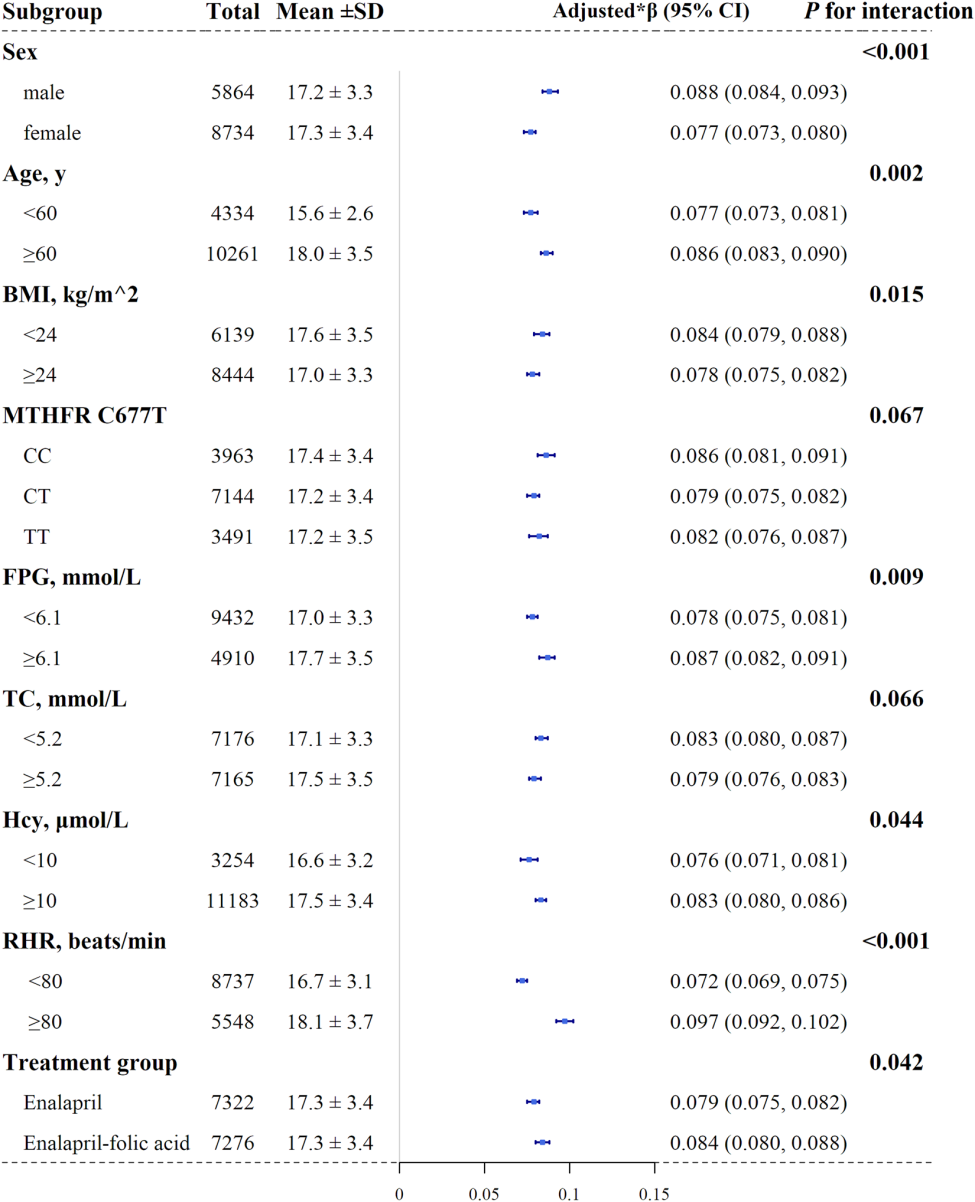
Subgroup analyses of the effect of SBP on baPWV. Abbreviations: baPWV, brachial–ankle pulse wave velocity; SBP, systolic blood pressure; BMI, body mass index; *MTHFR*, methylenetetrahydrofolate reductase; FPG, fasting plasma glucose; TC, total cholesterol; Hcy, homocysteine; RHR, resting heart rate. *Adjusted for age, sex, center, MTHFR C677T polymorphisms, treatment group, antihypertensive treatment, BMI, smoking status, and alcohol consumption, RHR, TC, HDL-cholesterol, TG, FPG, creatinine, hcy, uric acid, if not be stratified.

The role of other covariables on the association between BP indices and baPWV was further explored. Figure 3 shows the results of a subgroup analysis assessing the association of baPWV with SBP. Regardless of subgroup, SBP was positively associated with baPWV, and the effect was more significant in the following subgroups: male group (male: β = 0.088, 95%CI: 0.084–0.093; female: β = 0.077, 95%CI: 0.073–0.080, *P* for interaction < 0.001), aged ≥60 years group (<60 years: β = 0.077, 95%CI: 0.073–0.081; ≥60 years: β = 0.086, 95%CI: 0.083–0.090, *P* for interaction = 0.002), and in the BMI < 24 kg/m^2^ group (<24 kg/m^2^: β = 0.084, 95%CI: 0.079–0.088; ≥24 kg/m^2^: β = 0.078, 95%CI: 0.075–0.082, *P* for interaction = 0.015), FPG ≥ 6.1 mmol/L group (<6.1 mmol/L: β = 0.078, 95%CI: 0.075–0.081; ≥6.1 mmol/L: β = 0.087, 95%CI: 0.082–0.091, *P* for interaction = 0.009), hcy ≥10 μmol/L group (<10 μmol/L: β = 0.076, 95%CI: 0.071–0.081; ≥10 μmol/L: β = 0.083, 95%CI: 0.080–0.086, *P* for interaction = 0.044), RHR ≥ 80 beats per minute (bpm) group (<80 bpm: β = 0.072, 95%CI: 0.069–0.075; ≥80 bpm: β = 0.097, 95%CI: 0.092–0.102, *P* for interaction < 0.001) and enalapril-folic acid group (enalapril: β = 0.079, 95%CI: 0.075–0.082; β = 0.084, 95%CI, 0.080–0.088, *P* for interaction = 0.042). However, the effect of SBP on baPWV was consistent within *MTHFR* C677T and TC groups (all *P* for interaction > 0.05).

**Figure 4:**
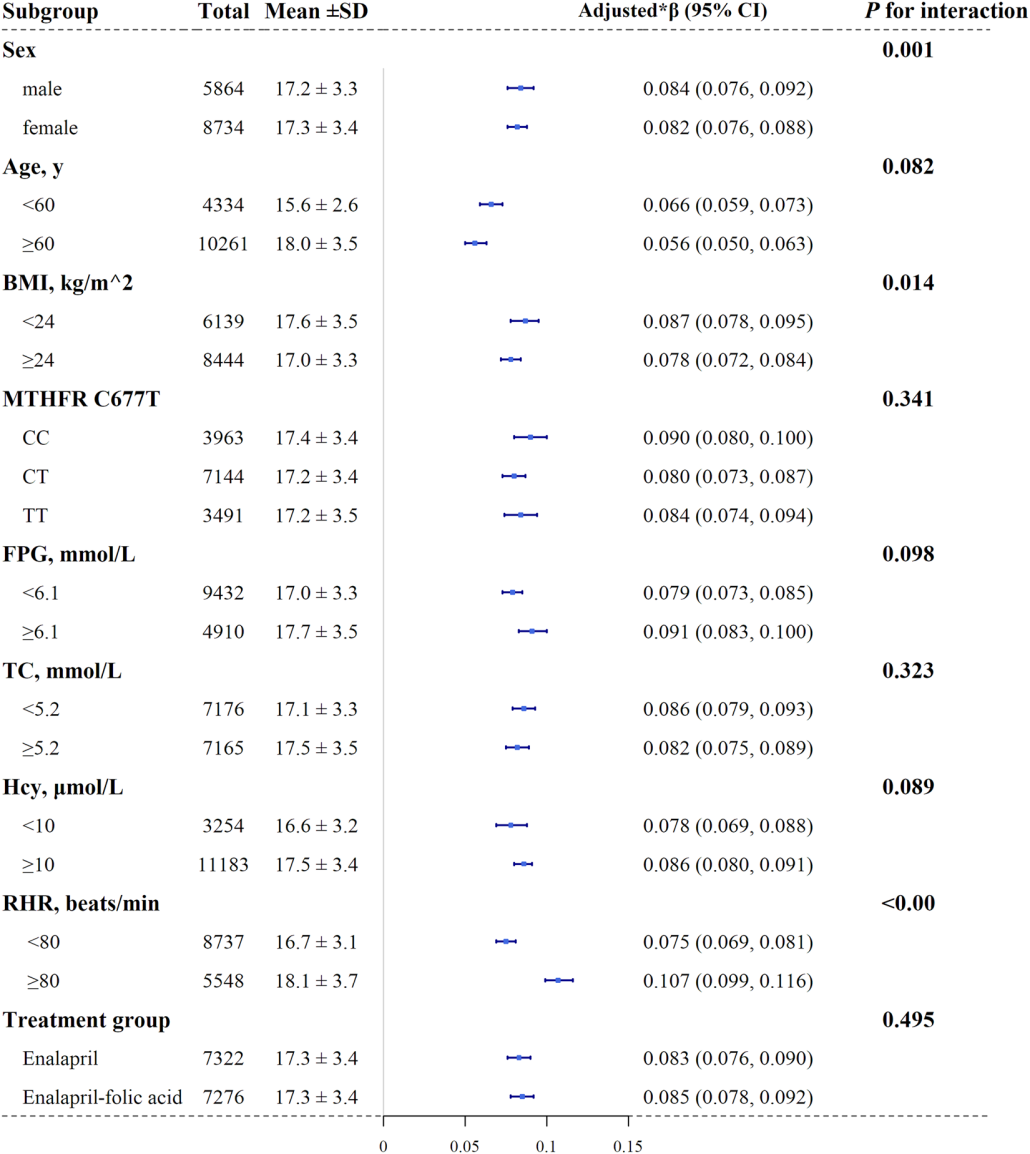
Subgroup analyses of the effect of DBP on baPWV. Abbreviations: baPWV, brachial–ankle pulse wave velocity; DBP, diastolic blood pressure; BMI, body mass index; *MTHFR*, methylenetetrahydrofolate reductase; FPG, fasting plasma glucose; TC, total cholesterol; Hcy, homocysteine; RHR, resting heart rate. *Adjusted for age, sex, center, MTHFR C677T polymorphisms, treatment group, antihypertensive treatment, BMI, smoking status, and alcohol consumption, RHR, TC, HDL-cholesterol, TG, FPG, creatinine, hcy, uric acid, if not be stratified.

Figure 4 shows that DBP was positively associated with baPWV in each subgroup; thus this association was more significant in male patients than that in female patients and in the BMI < 24 kg/m^2^ group than that in the BMI ≥ 24 kg/m^2^ group and in RHR ≥ 80 bpm group (male: β = 0.084, 95%CI: 0.076–0.092; female: β = 0.082, 95%CI: 0.076–0.088, *P* for interaction = 0.001; BMI < 24 kg/m^2^: β = 0.087, 95%CI: 0.078–0.095; ≥24 kg/m^2^: β = 0.078, 95%CI: 0.072–0.084, *P* for interaction = 0.014; <80 bpm: β = 0.075, 95%CI: 0.069–0.081; ≥80 bpm: β = 0.107, 95%CI: 0.099–0.116, *P* for interaction < 0.001).

Supplementary Figure 1 shows the results of a subgroup analysis assessing the association of baPWV with PP. PP was also positively associated with baPWV in each subgroup. The stronger association between PP and baPWV were detected in high FPG (≥6.1 mmol/L) higher RHR (≥80 bpm) and enalapril-folic acid group (all *P* for interaction < 0.05). As with SBP, the positive association between MAP and baPWV was more stronger in males, age ≥60 years, BMI < 24 kg/m^2^, FPG ≥ 6.1 mmol/L, hcy ≥ 10 μmol/L and RHR ≥ 80 bpm groups compared with their corresponding groups (all *P* for interaction < 0.05) (Supplementary Fig. 2).

## Discussion

In the present study, we found that SBP, DBP, PP and MAP levels were independently, significantly and positively associated with baPWV and elevated baPWV in treated hypertensive populations. The fully-adjusted smooth curve fitting showed a linear association between BP indices with baPWV. Moreover, uncontrolled SBP and DBP were significantly associated with higher baPWV. These findings suggest that baPWV might be a way to predict uncontrolled BP.

To our knowledge, few small sample population-based studies have addressed the relationship between BP indices and baPWV in treated hypertensive populations. Meili Zheng *et al*.^20^ used data from 3056 treated hypertensive subjects and found that baPWV appeared to be an independent determinant of individual response to anti-hypertensive treatment, most notably for SBP (with estimated coefficients of −9.01 for the top quartile, as compared to the bottom quartile). Protogerou Athanase *et al*.^21^ conducted a randomized, double-blind trial of 375 patients with hypertension and concluded that baseline PWV was a significant predictor of BP response to antihypertensive treatment, independent from age. Another study in patients with resistant hypertension indicated that a lower PWV reflected the predictors of higher BP reduction during spironolactone treatment^22^. Coutinho *et al*.^23^ noted that PWV was associated with longitudinal increases in SBP, MAP and PP but not DBP in a community-based cohort of hypertensive individuals. Consistent with previous studies, our study included a large sample and further showed that BP indices (SBP, DBP, MAP and PP) were independently and positively associated with baPWV and elevated baPWV in hypertensive populations. We also found the linear relationship between BP indices and baPWV using the smooth curve fitting. Moreover, uncontrolled SBP and DBP were significantly associated with higher baPWV. The study raises the possibility that baPWV may serve as a simple and noninvasive measurement to identify hypertensive adults whose BP was not being well controlled and a novel therapeutic target to further reduce CVD risk. Further research is needed to clarify the issue.

According to STROBE statement^24^, subgroup analysis can make better use of data to reveal underlying truths. Our findings showed that BP indices were positively assoiated with baPWV in subgroups and some fators modified the associations. The results suggested that males showed the stronger association between BP indices and baPWV. Some potential mechanisms including sex hormones could help explain the sex differences in this association. Male was a common risk factor for hypertension and arterial stiffness^25,26^. In aging males, hypogonadism is associated with increased arterial stiffening in central and peripheral vessels, while testosterone replacement reduces PWV^27^. In addition, we found that age could modify the associations of baPWV with SBP and MAP. Some researchers have confirmed that arterial stiffness and BP are increasing with the aging process^28,29^. Shouling Wu *et al*. also found that arterial stiffness mediated the positive association between aging and BP^13^. However, although our results showed that the association between DBP and baPWV was weaker in elderly, there was no statistical difference in the interaction test between different age groups. Perhaps because DBP decreased with increasing age, which was a major determinant of increasing baPWV^30,31^. Interestingly, we found that the effect of BP indices on baPWV was more significant in the low BMI group, suggesting a negative relationship between BMI and baPWV^32,33^. This finding might be due to the characteristics of our study participants: the majority of whom were farmers. We suspected that BMI in this population might reflect muscle mass and physical activity rather than adiposity. This finding was also in accordance with prior research^34,35^. Compared to the findings of previous studies^25,32,36^, we found, moreover, that higher RHR still had a higher association between baPWV and BP indices, suggesting RHR was the common risk factor for BP and baPWV. This phenomenon might be related to sympathetic nerve activity. Furthermore, previous research reported that FPG and hcy were independent risk factors for baPWV^11,26^, the present study investigated a large community-based sample from China and further confirmed that those with the higher FPG and hcy had a stronger association between baPWV and SBP and MAP.

### Limitations and strengths

Several limitations of the study are worth mentioning. This study was cross-sectional and failed to establish a cause-and-effect relationship between BP indices and baPWV. Additionally, the study participants were from a Chinese, rural hypertensive population aged 45 to 75 years old; thus, the generalizability of the results to other populations remained to be verified. Despite these limitations, this study was one of the largest to assess the associations between BP indices and baPWV in hypertensive populations. Our study also reported the linearity between BP indices and baPWV using smooth curve fitting (penalized spline method).

## Conclusions

In summary, our study showed that BP indices (including SBP, DBP, PP and MAP) were independently and positively associated with baPWV in treated hypertensive patients. Moreover, linearity between BP indices with baPWV was found. These findings suggest that baPWV might be a way to predict uncontrolled BP in treated hypertensive patients.

## Supplementary information

Supplement figures
