# Supplementary material for: Associations between Blood Pressure Indices and Brachial–ankle Pulse Wave Velocity in Treated Hypertensive Adults: results from the China Stroke Primary Prevention Trial (CSPPT)
Source: Sci Rep. 2019 Jun 3;9:8178. doi: 10.1038/s41598-019-44740-z (PMC6547652; doi:10.1038/s41598-019-44740-z)
Supplement: Supplementary file 1 — Supplement figures [file 41598_2019_44740_MOESM1_ESM.pdf]

Associations between Blood Pressure Indices and Brachial–ankle Pulse Wave Velocity in Treated Hypertensive Adults: results from the China Stroke Primary Prevention Trial (CSPPT)

Lihua Hu[1], M.D., Yuanyuan Zhang[2], M.D., Xiao Huang[1], M.D., Ph.D., Yun Song[2], M.D., Xianhui Qin[2,3], M.D., Binyan Wang[2,3], M.D., Ph.D., Yan Zhang[4], M.D., Genfu Tang[5], M.D., Jianping Li[4], M.D., Ping Li[1], M.D., Ph.D., Huihui Bao[1]*, M.D., Ph.D., Yong Huo[4], M.D., Xiaoshu Cheng[1]*, M.D., Ph.D.

[1]Department of Cardiovascular Medicine, the Second Affiliated Hospital of Nanchang University, Nanchang of Jiangxi, China.

[2]National Clinical Research Study Center for Kidney Disease; State Key Laboratory for Organ Failure Research; Renal Division, Nanfang Hospital, Southern Medical University, Guangzhou, China.

[3]Institute of Biomedicine, Anhui Medical University, Hefei, China.

[4]Department of Cardiology and Heart Center, Peking University First Hospital, Beijing, China.

[5]School of Health Administration, Anhui University, Hefei, China.

*** Correspondence and reprint requests should be addressed to:**

Huihui Bao, M.D., Ph.D.

Department of Cardiovascular Medicine, the Second Affiliated Hospital of Nanchang University, Nanchang of Jiangxi, China.

Email: huihui_bao77@126.com

Phone: +8613870092915

Fax: 0086-0791-86262262

or

Xiaoshu Cheng, M.D., Ph.D.

Department of Cardiovascular Medicine, the Second Affiliated Hospital of Nanchang University, Nanchang of Jiangxi, China.

Email: xiaoshumenfan126@163.com

Phone: +8613607089128

Fax: 0086-0791-86262262

Supplementary figure 1. Subgroup analyses of the effect of PP on baPWV.

Subgroup	Total	Mean ±SD	Adjusted*β (95% CI)	*P* for interaction
**Sex**				**0.096**
male	5864	17.2 ± 3.3	0.085 (0.080, 0.091)	
female	8734	17.3 ± 3.4	0.073 (0.069, 0.077)	
**Age, y**				**0.389**
<60	4334	15.6 ± 2.6	0.085 (0.080, 0.091)	
≥60	10261	18.0 ± 3.5	0.087 (0.083, 0.091)	
**BMI, kg/m^2**				**0.58**
<24	6139	17.6 ± 3.5	0.078 (0.073, 0.083)	
≥24	8444	17.0 ± 3.3	0.077 (0.073, 0.081)	
**MTHFR C677T**				**0.086**
CC	3963	17.4 ± 3.4	0.084 (0.078, 0.091)	
CT	7144	17.2 ± 3.4	0.074 (0.070, 0.079)	
TT	3491	17.2 ± 3.5	0.077 (0.070, 0.083)	
**FPG, mmol/L**				**0.047**
<6.1	9432	17.0 ± 3.3	0.075 (0.071, 0.079)	
≥6.1	4910	17.7 ± 3.5	0.082 (0.076, 0.088)	
**TC, mmol/L**				**0.118**
<5.2	7176	17.1 ± 3.3	0.080 (0.075, 0.084)	
≥5.2	7165	17.5 ± 3.5	0.075 (0.071, 0.080)	
**Hcy, µmol/L**				**0.576**
<10	3254	16.6 ± 3.2	0.074 (0.068, 0.081)	
≥10	11183	17.5 ± 3.4	0.078 (0.074, 0.082)	
**RHR, beats/min**				**<0.001**
<80	8737	16.7 ± 3.1	0.068 (0.064, 0.072)	
≥80	5548	18.1 ± 3.7	0.086 (0.080, 0.092)	
**Treatment group**				**0.016**
Enalapril	7322	17.3 ± 3.4	0.073 (0.068, 0.077)	
Enalapril-folic acid	7276	17.3 ± 3.4	0.082 (0.078, 0.087)	

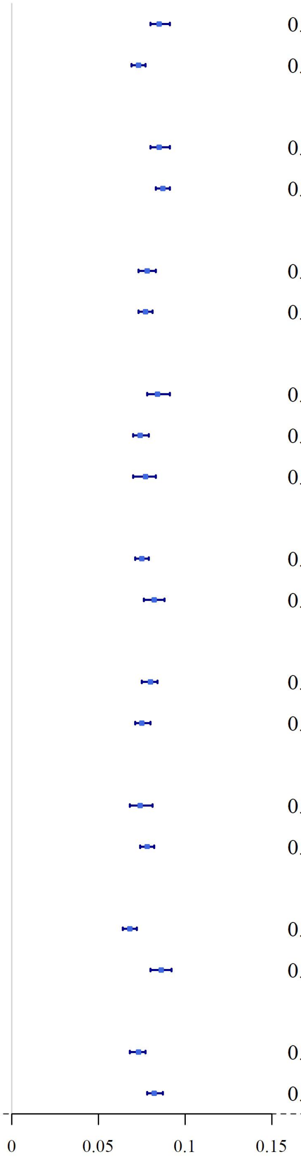

Abbreviations: baPWV, brachial–ankle pulse wave velocity; PP, pulse pressure; BMI, body mass index; *MTHFR*, methylenetetrahydrofolate reductase; FPG, fasting plasma glucose; TC, total cholesterol; Hcy, homocysteine; RHR, resting heart rate.

**adjusted for age, sex, center, MTHFR C677T polymorphisms, treatment group, antihypertensive treatment, BMI, smoking status, and alcohol consumption, RHR, TC, HDL-C, TG, FPG, creatinine, hcy, uric acid, if not be stratified.*

Supplementary figure 2. Subgroup analyses of the effect of MAP on baPWV.

Subgroup	Total	Mean ±SD	Adjusted*β (95% CI)	*P* for interaction
**Sex**				**0.001**
male	5864	17.2 ± 3.3	0.118 (0.111, 0.125)	
female	8734	17.3 ± 3.4	0.111 (0.106, 0.117)	
**Age, y**				**0.009**
<60	4334	15.6 ± 2.6	0.094 (0.088, 0.100)	
≥60	10261	18.0 ± 3.5	0.107 (0.101, 0.113)	
**BMI, kg/m^2**				**<0.001**
<24	6139	17.6 ± 3.5	0.120 (0.113, 0.127)	
≥24	8444	17.0 ± 3.3	0.107 (0.102, 0.113)	
**MTHFR C677T**				**0.196**
CC	3963	17.4 ± 3.4	0.121 (0.112, 0.129)	
CT	7144	17.2 ± 3.4	0.111 (0.104, 0.117)	
TT	3491	17.2 ± 3.5	0.115 (0.107, 0.124)	
**FPG, mmol/L**				**0.009**
<6.1	9432	17.0 ± 3.3	0.109 (0.103, 0.114)	
≥6.1	4910	17.7 ± 3.5	0.123 (0.116, 0.131)	
**TC, mmol/L**				**0.111**
<5.2	7176	17.1 ± 3.3	0.118 (0.111, 0.124)	
≥5.2	7165	17.5 ± 3.5	0.112 (0.105, 0.118)	
**Hcy, µmol/L**				**0.014**
<10	3254	16.6 ± 3.2	0.106 (0.098, 0.114)	
≥10	11183	17.5 ± 3.4	0.117 (0.112, 0.122)	
**RHR, beats/min**				**<0.001**
<80	8737	16.7 ± 3.1	0.102 (0.097, 0.108)	
≥80	5548	18.1 ± 3.7	0.138 (0.131, 0.146)	
**Treatment group**				**0.282**
Enalapril	7322	17.3 ± 3.4	0.113 (0.106, 0.119)	
Enalapril-folic acid	7276	17.3 ± 3.4	0.117 (0.111, 0.123)	

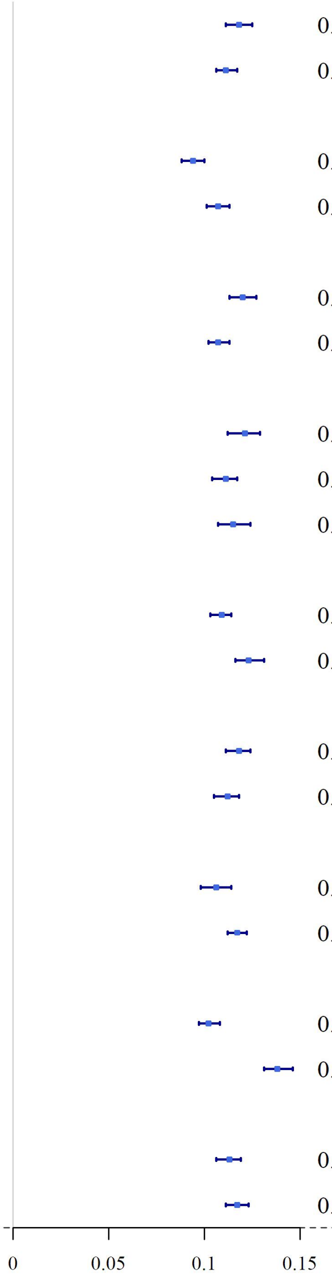

Abbreviations: baPWV, brachial–ankle pulse wave velocity; MAP, mean arterial pressure; BMI, body mass index; *MTHFR*, methylenetetrahydrofolate reductase; FPG, fasting plasma glucose; TC, total cholesterol; Hcy, homocysteine; RHR, resting heart rate.

**adjusted for age, sex, center, MTHFR C677T polymorphisms, treatment group, antihypertensive*

treatment, BMI, smoking status, and alcohol consumption, RHR, TC, HDL-C, TG, FPG, creatinine, hcy, uric acid, if not be stratified.
